# Disability Transitions and Health Expectancies among Elderly People Aged 65 Years and Over in China: A Nationwide Longitudinal Study

**DOI:** 10.14336/AD.2019.0121

**Published:** 2019-12-01

**Authors:** Chengbei Hou, Yuan Ma, Xinghua Yang, Lixin Tao, Deqiang Zheng, Xiangtong Liu, Xiaonan Wang, Xia Li, Wei Wang, Xianghua Fang, Xiuhua Guo

**Affiliations:** ^1^School of Public Health, Capital Medical University, Beijing, China.; ^2^Beijing Municipal Key Laboratory of Clinical Epidemiology, School of Public Health, Capital Medical University, Beijing, China; ^3^Department of Mathematics and Statistics, La Trobe University, Bundoora Victoria, Australia.; ^4^School of Medical Sciences and Health, Edith Cowan University, Joondalup, Perth, WA6027, Australia.; ^5^Center for Evidence-Based Medicine, Xuanwu Hospital Capital Medical University, Beijing, China

**Keywords:** disability transitions, health life expectancy, multistate model, basic activities, daily living, instrumental activities

## Abstract

Disability has become a critical issue among elderly populations, yet limited large-scale research related to this issue has been conducted in China, an aging society. This study explored sex and urban-rural differences in disability transitions and life expectancies among older adults in China. Data were collected from the Chinese Longitudinal Health Longevity Survey (CLHLS), which enrolled people aged 65 and older and was conducted in randomly selected counties and cities across 22 provinces in China. Disability was diagnosed based on basic activities of daily living (BADLs) and instrumental activities of daily living (IADLs). Several individual characteristics were assessed, including sociodemographic factors (age, sex and region, etc.) and health behaviors (currently smoking, currently drinking, etc.). Multistate models were applied to analyze the transition rates among 4 states: no disability, mild disability, severe disability and death. The transition rates from disabled states to the no-disability state were found to decrease markedly with age. The rates of recovery from mild disability in rural areas were higher than those in urban areas. Rural elderly individuals lived shorter lives than their urban counterparts, but they tended to live with better functional status, spending a larger fraction of their remaining life with less severe disability. Based on these findings, devoting more attention and resources to rural areas may help less severely disabled people recuperate and prevent severe disability. The study provides insights into health plan strategies to help guide the allocation of limited resources.

Functional disability is prevalent in older adults, and it is associated with a considerable loss of independence, reductions in quality of life and even death [1-3], and the proportion and severity of disability increase with age [4-6]. In addition to increasing the cost of health care, functional decline may impose a burden of substantial uncompensated informal care on their families [7-9]. China has the largest number of aged citizens and a high proportion of the aged population in the world, and accelerated growth of the aging population will continue in China in the coming decades [10-14], which will unavoidably lead to serious challenges to its medical resources and health services.

The rapid rise in disabled elderly populations in society has become an increasingly prominent issue that warrants greater attention. Health life expectancy, defined as the average remaining lifetime in different health states, is necessary for national health policy establishment, health program evaluation and health promotion. Studies have begun to examine health expectancies and to analyze differences in race and sex [15-19]. Using data from the LSOA II, Hagedorn A [19] examined active life expectancies and showed similar sex differences in the proportion of active life expectancy to total life expectancy at age 70 among populations in the United States and Japan. A study based on the INDEPTH WHO-SAGE revealed how different socioeconomic indicators such as education, marital status, living arrangement and household socioeconomic status influence the health inequality observed between men and women in eight countries in Africa and Asia [20]. Studies have also examined rural and urban differences in health issues [21-24]. Laditka et al. [23] found that the elderly in rural areas may experience a longer expected period of impairment than the elderly in urban areas.

There have also been several studies related to the disability and life expectancy of elderly Chinese individuals [23, 25]. A national study conducted in 2010 showed that the prevalence of elderly with disabilities was 6.25% [26]. Several studies have indicated that factors such as residence [27, 28] and household structure [29, 30] impact disability. However, these studies were usually small in scale and conducted at the municipal level and are therefore of limited use for nationwide generalization. Although there have been some national studies on disability in elderly Chinese individuals, these studies typically did not investigate the factors associated with disability. In addition, most previous studies in this field are based on cross-sectional designs [31], which could not take into account the transition between different disabled states.

**Figure 1.**
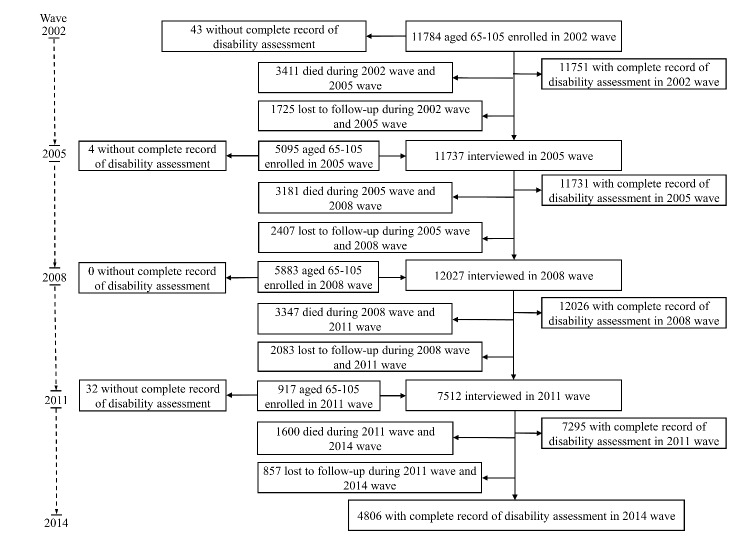
Flowchart of participant inclusion and follow-up.

To the best of our knowledge, there have been no studies involving urban-rural residence differences in health expectancy for elderly Chinese individuals. In this study, we hypothesized that among older adults in China, differences exist between urban and rural elderly individuals and between men and women in terms of their transitions among disabled states and in their expected years with no, mild, and severe disability.

## MATERIALS AND METHODS

### Study design and participants

The data used in this study were collected from the Chinese Longitudinal Health Longevity Survey (CLHLS), managed by the Center for Healthy Aging and Development Studies, Peking University. The CLHLS is a nationwide population-based longitudinal survey of the determinants of healthy aging conducted in randomly selected counties and cities across 22 provinces in China. The CLHLS was first launched in 1998 and then followed in 2000, 2002, 2005, 2008, 2011 and 2014. The first and second waves of the CLHLS were limited to persons aged 80 and above, and then people aged 65 to 79 were added to the survey in 2002 and in subsequent waves. At each follow-up wave, the surviving participants were re-interviewed, and new participants with the same age, sex, and region of residence were enrolled to account for attrition and death. The CLHLS study was conducted according to the guidelines established by the Declaration of Helsinki, and all procedures involving participants were approved by Duke University Health System’s Institutional Review Board members and the Biomedical Ethics Committee of Peking University (IRB00001052-13074). All subjects provided written informed consent to indicate their willingness to participate in the CLHLS. The informed consent form was signed by the next-of-kin in the case when the respondent was unable to sign. More details about the study design, sampling, measures, and data quality of the CLHLS are available elsewhere (https://sites.duke.edu/centerforaging/programs/chinese-longitudinal-healthy-longevity-survey-clhls/).

We focus on individuals aged 65 to 95 at the first observation point of this analysis (baseline) based on the last five waves from 2002 to 2014. In the 2002 wave, 11,784 people aged 65-95 were interviewed, and 5,095, 5,883 and 917 new participants were enrolled in 2005, 2008 and 2011, respectively. We excluded individuals who dropped out before their first follow-up (n=3,981), as well as those without a complete disability assessment at baseline (n=79). Ultimately, an analytical sample of 19,619 respondents was used (Fig. 1), for which 45.2%, 30.2%, 13.4%, and 11.2% had data (including death) in 2, 3, 4, and all 5 waves of the survey, respectively. Dates of death of the subjects before the subsequent wave were obtained from immediate family members. All information in each wave was collected by trained staff members from the county Centers for Disease Control and Prevention.

### Measures

Disability was based on basic activities of daily living (BADLs) and instrumental activities of daily living (IADLs). The following BADL items were surveyed in each wave: bathing, dressing, eating, toileting and indoor transferring. The following IADL items were surveyed: visiting neighbors, shopping for groceries, washing clothes, preparing meals and taking public transportation. Disabled states were defined as severe disability if the participant was unable to perform any one of five BADL items without help; mild disability if they could perform all BADL items without help from another person but needed help when performing any one of the five IADL items; and no disability if they were able to perform all BADLs and IADLs independently [32].

Individual characteristics that might influence disability, including sociodemographic characteristics and health behaviors, were assessed upon entry into the survey. Sociodemographic characteristics included age group (65-74 y/ 75-84 y/85-95 y), sex (male/female), region (rural/urban), and marital status (currently married/others: divorced, widowed, separated, or never married), educational attainment (0 year/1-6 years/7+ years of schooling), living expenses (sufficient/ insufficient), and medical services (adequate/inadequate). Health behaviors included regular exercise (yes/no), smoking status (yes/no), and alcohol consumption (yes/no). Self-reported chronic conditions included diabetes, cardiovascular disease, and stroke.

In the CLHLS, some questions were answered by the interviewees only, such as self-ratings of their health and life satisfaction and the questions from the Mini-Mental State Examination (MMSE). Other questions were answered by the interviewees as much as possible. For those who were unable to answer these questions, a close family member or another knowledgeable proxy (i.e., significant other) provided answers, as indicated previously. An indicator question was marked by the interviewer to signify whether the answer was provided by the interviewee or the proxy [33].

### Statistical analysis

Multistate models [34] were applied to analyze the transition rates among the 4 states: no disability, mild disability, severe disability and death. The transitions were modeled between disabled states and from any disabled state to death (Figure S1). The transition intensities between states were estimated using a proportional hazards regression model, assuming the instantaneous rate of transition was constant across the observed time intervals. Since the exact date of transitions between disabled states was not available, we assumed a continuous-time Markov process, which meant that the disabled status of a participant could change at any time within each survey interval.

Two separate statistical analyses were performed: multistate life table analyses and Cox proportional hazards analyses. For the transition probability and health expectancy, an age-, sex- and region-specific model was used to estimate the transition intensities in order to construct the multistate life table. The maximum likelihood estimator was used as a point estimation of the annual transition probabilities, and the 95% confidence interval (CI) was estimated by the bootstrap method with 499 resamples [35]. Based on Van de Hout and Matthews [36], the sex- and region-specific 65-, 75-, 85-, and 95-year-olds’ health expectancies and the 95% CIs were computed. In the computation, the initial distribution of status (except for death) in a certain group was estimated by empirical distribution. We combined mild and severe disabled states into one disability in order to compute the hazard ratio of disability onset. The association of disability with sociodemographic characteristics (sex, educational attainment, marital status, living expenses, and medical service), health behavior factors (currently smoking, currently drinking, and regular exercise), or chronic disease (diabetes, cardiovascular disease, and stroke) at the first entry into the survey was examined after controlling for potential confounders and, simultaneously, the other factors mentioned above.

Missing values were imputed using multiple imputation with a predictive mean matching method [37]. The multiple imputation models included age, sex, region, marital status, educational attainment, living expenses, medical service, regular exercise, smoking status, alcohol consumption, diabetes, cardiovascular disease, and stroke. Five imputed data points replacing each missing value with a set of plausible values were generated. We performed analyses for each of these five imputed data points and took the mean of the five results as the result of the multiple imputations.

**Table 1:** Demographic characteristics and health status of the participants.

Variables	Urban		Rural	
	Men (N=3838)	Women (N=3918)	Men (N=5866)	Women (N=5997)
Age, Mean±SD	82.0±9.2	83.0±9.2	81.7±9.3	83.2±9.4
Educational attainment, N (%)				
0 year	1023 (26.7)	2720 (69.8)	2378 (40.6)	5100 (85.4)
1-6 years	1810 (47.3)	837 (21.5)	2782 (47.5)	798 (13.4)
7-9 years	417 (10.9)	171 (4.4)	420 (7.2)	49 (0.8)
9 + years	577 (15.1)	167 (4.3)	272 (4.6)	25 (0.4)
Currently married, N (%)	2142 (55.8)	946 (24.1)	2932 (50.0)	1504 (25.1)
Currently smoking, N (%)	1260 (32.9)	325 (8.3)	2351 (40.1)	418 (7.0)
Currently drinking, N (%)	1169 (30.5)	355 (9.1)	2101 (35.8)	597 (10.0)
Sufficient living expenses, N (%)	3269 (85.2)	3117 (79.6)	4538 (77.4)	4532 (75.6)
Adequate medical service, N (%)	3623 (94.4)	3608 (92.1)	5252 (89.5)	5266 (87.8)
Physical exercise, N (%)	2028 (52.9)	1490 (38.1)	1600 (27.3)	1124 (18.8)
Diabetes, N (%)	156 (4.1)	158 (4.0)	72 (1.2)	102 (1.7)
Stroke, N (%)	309 (8.1)	249 (6.4)	294 (5.0)	264 (4.4)
Cardiovascular disease, N (%)	457 (11.9)	559 (14.3)	307 (5.2)	395 (6.6)
Disability at entry, N (%)				
No disability	2145 (55.9)	1574 (40.2)	3169 (54.0)	2260 (37.7)
Mild disability	1064 (27.7)	1464 (37.4)	2042 (34.8)	2702 (45.1)
Severe disability	629 (16.4)	880 (22.5)	655 (11.2)	1035 (17.3)

Note: Data come from the Chinese Longitudinal Healthy Longevity Survey (CLHLS). Numbers calculated as a percentage of the nonmissing values. The number of participants with missing values at entry was 73 for education, 2 for marital status, 14 for currently smoking, 14 for currently drinking, 4 for living expenses, 4 for medical service, 30 for physical activity, 6 for diabetes, 4 for cardiovascular disease, and 5 for stroke.

Statistical analyses were conducted using R version 3.3.2 (R Foundation for Statistical Computing, Vienna, Austria).

## RESULTS

### Characteristics and health status of the participants

The characteristics of the elderly participants in our study stratified by urban-rural residential region and sex are shown in Table 1. Males and females were almost equal in number for both the urban and rural areas. The mean age of the urban participants was 82.0±9.2 years for men and 83.0±9.2 years for women, which was close to that of rural participants (81.7±9.3 years for men and 83.2±9.4 years for women). A higher proportion of the urban participants received a high level of education and were married, and a higher proportion of the rural participants currently smoked and drank. A greater proportion of urban elderly participants had sufficient finances to offset living expenses (82.4% versus 76.5%) and received adequate medical services (93.4% versus 88.6%) compared to rural elderly participants. Physical exercise was significantly associated with urban-rural residential regions, with 45.4% of people engaging in physical exercise among urban elderly participants, while the proportion was only 23.0% for rural elderly participants. The prevalence of disability was high (52.1% and 54.4% for urban elderly and rural elderly, respectively) in this population.

**Figure 2.**
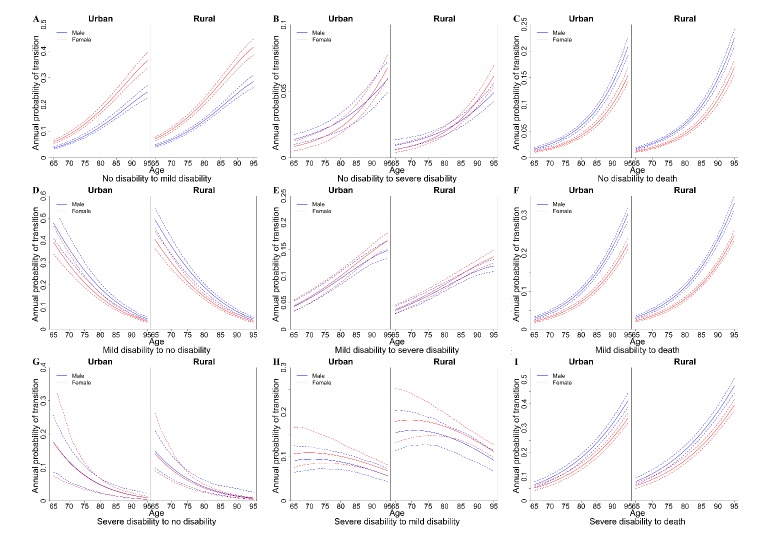
Estimated annual transition probabilities by age. (A) The annual probability of transition from no disability to mild disability. (B) The annual probability of transition from no disability to severe disability. (C) The annual probability of transition from no disability to death. (D) The annual probability of transition from mild disability to no disability. (E) The annual probability from mild disability to severe disability. (F) The annual probability from mild disability to death. (G) The annual probability of transition from severe disability to no disability. (H) The annual probability of transition from severe disability to mild disability. (I) The annual probability of transition from severe disability to death.

**Table 2:** Hazard ratios by risk factors upon onset of disability.

No impaired to any impaired	Model1	Model2	Model3	Model4
	HR (95%CI)	HR (95%CI)	HR (95%CI)	HR (95%CI)
Gender				
Male	1.00	1.00	1.00	1.00
Female	1.27(1.20~1.35)	1.43(1.32~1.54)	1.17(1.07~1.29)	1.15(1.05~1.27)
Educational attainment				
0 year	1.00	1.00	1.00	1.00
1-6 years	0.65(0.60~0.69)	0.79(0.72~086)	0.79(0.72~0.86)	0.79(0.72~0.86)
7+ years	0.44(0.40~0.49)	0.61(0.55~0.71)	0.61(0.53~0.70)	0.62(0.54~0.70)
Marital status				
Others	1.00	1.00	1.00	1.00
Currently married	0.59(0.55~0.63)	0.98(0.90~1.06)	1.00(0.92~1.08)	1.00(0.92~1.08)
Currently smoking				
No	1.00	1.00	1.00	1.00
Yes	0.74(0.69~0.80)	0.94(0.85~1.03)	0.92(0.84~1.01)	0.91(0.83~1.01)
Currently drinking				
No	1.00	1.00	1.00	1.00
Yes	0.80(0.74~0.85)	0.94(0.85~1.03)	0.95(0.86~1.04)	0.95(0.86~1.04)
Regular exercise				
No	1.00	1.00	1.00	1.00
Yes	0.84(0.78~0.89)	0.89(0.82~0.96)	0.93(0.86~1.01)	0.93(0.86~1.01)
Living expenses				
Not enough	1.00	1.00	1.00	1.00
Enough	0.89(0.83~0.96)	0.94(0.86~1.03)	0.99(0.89~1.09)	0.99(0.90~1.10)
Medical service				
Not adequate	1.00	1.00	1.00	1.00
Adequate	0.74(0.66~0.83)	0.88(0.76~1.01)	0.91(0.78~1.06)	0.93(0.80~1.08)
Diabetes				
No	1.00	1.00	1.00	1.00
Yes	0.85(0.69~1.05)	0.94(0.76~1.18)	0.94(0.75~1.18)	0.89(0.71~1.12)
Heart disease				
No	1.00	1.00	1.00	1.00
Yes	0.94(0.84~1.04)	1.14(1.00~1.31)	1.17(1.02~1.35)	1.18(1.02~1.37)
Stroke				
No	1.00	1.00	1.00	1.00
Yes	1.31(1.13~1.53)	1.27(1.08~1.49)	1.24(1.05~1.46)	1.22(1.03~1.44)

Note: Data come from the Chinese Longitudinal Healthy Longevity Survey (CLHLS). Model1 was univariate; Model2 was controlled for age, gender and region; Model3 was additionally controlled for education attainment, marital status, currently smoking, currently drinking, regular exercise, living expenses, and medical service. Model 4 was further controlled for disease factors: diabetes, stroke and heart disease. When these factors were considered as covariates in analyses, a dummy variable for each of these factors was created to represent the group of subjects with the missing value.

### Factors associated with disability

The association of disability with each sociodemographic or health behavior factor was examined (Table 2). Higher educational attainment was shown to be a protective factor (HR=0.79 for 1-6 years of education, HR=0.62 for 7+ years of education). Compared with elderly men, elderly women were 1.15 times more likely to have some level of disability. In addition, elderly individuals who suffered from stroke or cardiovascular disease had 0.22 and 0.18 higher probabilities of disability, respectively.

**Table 3:** Total life expectancy and proportion of remaining life with or without disability.

	Urban		Rural	
Age	Male	Female	Male	Female
65				
TLE, years (95% CI) ND, years (95% CI) PND, proportion (95% CI) AD, years (95% CI) PAD, proportion (95% CI) MD, years (95% CI) PMD, proportion (95% CI) SD, years (95% CI) PSD, proportion (95% CI) 75 TLE, years (95% CI) ND, years (95% CI) PND, proportion (95% CI) AD, years (95% CI) PAD, proportion (95% CI) MD, years (95% CI) PMD, proportion (95% CI) SD, years (95% CI) PSD, proportion (95% CI)	14.92 (14.53,15.32)	17.02 (16.66,17.46)	14.69 (14.32,15.04)	16.48 (16.14,16.83)
	10.86 (10.51,11.21)	10.59 (10.27,10.91)	10.83 (10.51,11.10)	10.00 (9.71,10.27)
	72.75 (71.32,74.04)	62.20 (60.85,63.50)	73.74 (72.74,74.73)	60.64 (59.42,61.81)
	4.07 (3.85,4.30)	6.43 (6.17,6.72)	3.86 (3.67,4.04)	6.49 (6.25,6.74)
	27.25 (25.96,28.68)	37.80 (36.50,39.15)	26.26 (25.27,27.26)	39.36 (38.19,40.58)
	2.53 (2.38,2.70)	4.46 (4.24,4.71)	2.90 (2.75,3.04)	5.09 (4.87,5.31)
	16.96 (16,17.92)	26.21 (25.15,27.49)	19.72 (18.87,20.55)	30.89 (29.85,31.99)
	1.53 (1.37,1.70)	1.97 (1.82,2.13)	0.96 (0.87,1.06)	1.40 (1.30,1.51)
	10.29 (9.12,11.41)	11.59 (10.64,12.53)	6.54 (5.92,7.23)	8.47 (7.91,9.18)
	8.74 (8.53,8.97)	10.04 (9.80,10.30)	8.41 (8.23,8.62)	9.57 (9.36,9.78)
	5.18 (5.00,5.37)	4.38 (4.23,4.54)	4.91 (4.76,5.05)	3.96 (3.83,4.09)
	59.18 (57.59,60.61)	43.65 (42.35,44.89)	58.38 (57.22,59.57)	41.35 (40.26,42.47)
	3.57 (3.41,3.73)	5.65 (5.46,5.87)	3.50 (3.36,3.64)	5.61 (5.45,5.79)
	40.82 (39.39,42.41)	56.35 (55.11,57.65)	41.62 (40.43,42.78)	58.65 (57.53,59.74)
	2.22 (2.11,2.35)	3.72 (3.57,3.90)	2.6 (2.50,2.72)	4.24 (4.10,4.39)
	25.39 (24.20,26.67)	37.06 (35.80,38.42)	30.95 (29.92,32.02)	44.3 (43.26,45.37)
	1.35 (1.23,1.46)	1.94 (1.82,2.06)	0.90 (0.83,0.96)	1.37 (1.29,1.47)
	15.43 (14.13,16.71)	19.29 (18.12,20.47)	10.67 (9.94,11.39)	14.35 (13.56,15.28)
85				
TLE, years (95% CI) ND, years (95% CI) PND, proportion (95% CI) AD, years (95% CI) PAD, proportion (95% CI) MD, years (95% CI) PMD, proportion (95% CI) SD, years (95% CI) PSD, proportion (95% CI)	4.23 (4.11,4.37)	5.09 (4.93,5.25)	4.11 (4.00,4.22)	4.84 (4.73,4.96)
	1.46 (1.39,1.54)	1.07 (1.01,1.12)	1.48 (1.42,1.54)	0.99 (0.95,1.04)
	34.45 (32.86,35.94)	20.99 (19.86,21.96)	35.93 (34.71,37.28)	20.42 (19.57,21.35)
	2.78 (2.66,2.90)	4.02 (3.88,4.18)	2.63 (2.54,2.72)	3.85 (3.74,3.96)
	65.55 (64.06,67.14)	79.01 (78.04,80.14)	64.07 (62.72,65.29)	79.58 (78.65,80.43)
	1.55 (1.47,1.62)	2.32 (2.21,2.43)	1.88 (1.80,1.96)	2.68 (2.59,2.78)
	36.58 (34.97,38.1)	45.62 (44.16,47.23)	45.74 (44.4,47.17)	55.45 (54.28,56.68)
	1.23 (1.15,1.31)	1.7 (1.61,1.81)	0.75 (0.69,0.81)	1.17 (1.11,1.23)
	28.97 (27.25,30.67)	33.39 (31.88,35.05)	18.33 (17.16,19.42)	24.13 (23.00,25.35)
95				
TLE, years (95% CI) ND, years (95% CI) PND, proportion (95% CI) AD, years (95% CI) PAD, proportion (95% CI) MD, years (95% CI) PMD, proportion (95% CI) SD, years (95% CI) PSD, proportion (95% CI)	1.89 (1.79,1.99)	2.35 (2.24,2.48)	1.73 (1.66,1.81)	2.21 (2.12,2.31)
	0.27 (0.25,0.29)	0.14 (0.13,0.16)	0.23 (0.21,0.25)	0.13 (0.12,0.14)
	14.16 (12.95,15.66)	5.99 (5.34,6.61)	13.09 (12.15,14.2)	5.89 (5.41,6.37)
	1.62 (1.53,1.72)	2.21 (2.10,2.34)	1.51 (1.43,1.58)	2.08 (1.99,2.18)
	85.84 (84.34,87.05)	94.01 (93.39,94.66)	86.91 (85.8,87.85)	94.11 (93.63,94.59)
	0.75 (0.70,0.80)	0.97 (0.90,1.03)	0.93 (0.88,0.99)	1.25 (1.18,1.32)
	39.92 (37.32,42.31)	41.16 (38.81,43.58)	53.89 (51.40,56.30)	56.4 (54.37,58.46)
	0.87 (0.79,0.95)	1.24 (1.16,1.35)	0.57 (0.52,0.62)	0.83 (0.77,0.90)
	45.92 (43.25,48.87)	52.85 (50.37,55.28)	33.02 (30.54,35.52)	37.71 (35.70,39.82)

Note: Data come from the Chinese Longitudinal Healthy Longevity Survey (CLHLS) TLE=total life expectancy; ND=years with no disability; MD=years with mild disability; SD=years with severe disability; AD=years with any disability (MD+SD); PND=proportion of life with no disability; PMD=proportion of life with mild disability; PSD=proportion of life with severe disability; PAD=proportion of life with any disability (PMD+PSD).

### Disability transitions

The study shows that the transition probability between disabled states differed by sex, age and residential region (Fig. 2). We found that women with no disability had a higher probability of obtaining a mild disability than their male counterparts, and the difference became greater with age (Fig. 2A), while men with any disabled status had a higher probability of death in both urban and rural areas (Fig. 2C, 2F, 2I). The transition rates from no disabled state or a mild disabled state to a worse disabled state rose sharply with age (Figure 2A). For example, urban women with no disability at 65 years old had a 5.9% (95% CI, 5.3-6.6%) probability of transferring to mild disability at age 66, but the annual probability rose to 17.8% (95% CI, 16.7-19.2%) when they reached 80 years old. Elderly adults in rural areas had a higher probability of transferring from no disability to mild disability (rural: 7.1% versus urban: 5.9% for 65-year-old women) but less probability of transferring from mild disability to severe disability than did urban old adults (rural: 3.7% versus urban: 4.4% for 65-year-old women).

In terms of disability improvement, the transition rates from mild and severe disability to the no disability state dropped markedly with age (Figure 2D, 2G). The probability of transition from severe disability to no disability was less than 5.0% after age 85. For instance, the full recovery probability was 2.6% from severe disability for 85-year-old urban men (Figure 2G). People in rural areas had greater point estimations for the probability of transitioning from mild disability to no disability and from severe disability to mild disability than did their urban counterparts (Fig. 2D, 2H).

### Health life expectancy

The expected remaining years of life for people with different disabled states at different ages are shown in Table 3. Women were expected to live longer than men and experienced a greater fraction of the remaining years with disability in both urban and rural areas. For example, at 65 years old, rural men were expected to live an additional 14.69 years (95% CI, 14.32-15.04), with the fraction of years with mild or severe disability accounting for 26.3% (95% CI, 25.3-27.3%), while their female counterparts were expected to live an additional 16.48 years (95% CI, 16.14-16.83), with the fraction of years with any level of disability accounting for 39.4% (95% CI, 38.2-40.6%).

A difference in health expectancy was also found between urban and rural areas. This finding indicated that, compared to their rural counterparts, 65-year-old men with no disability in urban areas were expected to live longer (14.92 years versus 14.69 years) but would spend a greater fraction of the remaining years with some level of disability (27.3% versus 26.3%). The fraction of remaining life years with disability was expected to increase with age for those in both urban and rural residential areas.

The results also reveal an important finding: people who have better initial functional states can live longer and spend a smaller fraction of their remaining life years suffering from disability (Table S1). For example, 65-year-old urban men with an initial state of mild disability will live 15.07 years (95% CI, 14.66-15.46), with a disability for 26.1% (95% CI, 24.8-27.4%) of their remaining life, while 65-year-old urban men with an initial state of severe disability will live 12.63 years (95% CI, 11.78-13.40), with a disability for 45.5% (95% CI, 40.1-51.8%) of their remaining life.

### Sensitivity analysis

No significant difference was found between the main analysis and multiple imputation analysis for the results of transition probabilities, life expectancies and proportion of life with any disabled state. The conclusions did not change after the imputation analyses were applied in the present study.

## DISCUSSION

In this large-scale national study, the transitions between disability states and health life expectancies among elderly people aged 65 years and over have been examined. We found that among older adults in China, differences exist between individuals living in urban regions and those living in rural regions and between men and women in terms of life expectancies and transition probabilities among different disabled states.

Women are known to have a longer life expectancy but a higher prevalence of disability for both eastern and western countries [37-44]. Our analysis verifies that women are expected to live longer and spend a larger proportion of their remaining years with disability than men. One explanation for this difference may be that men generally having a higher prevalence of life-threatening diseases, while women are more likely to develop diseases that do not result in death but may contribute negatively to the rates of functional limitation and disability, such as headache, asthma, arthritis, depression, and cognitive loss [42, 44, 45]. Health life expectancies are influenced by a complex combination of many factors, including biological factors (such as genetic and hormonal differences), environmental conditions and health behaviors [46, 47]. Several studies have indicated that at least three-quarters of sex differences in mortality were not due to biological factors [48-50]. Other studies have suggested that human actions have a great influence on the mortality gap between men and women [51, 52]. Studies have demonstrated that excess mortality in men was significantly lower in groups where men and women had homogeneous lifestyles and social environments, for example, among nonsmokers [53], Mormons [54], Seventh-Day Adventists [55], etc. Therefore, specific subpopulations of men with particularly high risk were the main cause of excess mortality [46, 47].

Similar to previous studies [56], our study revealed that rural elderly individuals lived shorter lives than their urban counterparts, which may be because medical services in rural regions are less accessible than those in urban regions [57, 58]. In addition, elderly individuals in rural areas are usually less engaged in regular physical exercise, which can protect people from cardiovascular diseases [59]. However, this study revealed that rural elderly individuals tend to live with better functional status than their urban counterparts and spend a larger proportion of their remaining life with less severe disability. This can be explained by the theory of survival selection [60]. Specifically, owing to poor medical accessibility in rural areas, “weak rural people” may be selectively eliminated from a surviving population, leaving those with hardier characteristics (e.g., unobserved genetic and behavioral characteristics) than their urban counterparts, resulting in a regional disparity for disability.

In addition to the factors mentioned above, lower education levels and chronic diseases such as stroke and cardiovascular disease are also risk factors for disability, which is consistent with other studies [61-63].

The major advantage of this study was that the study was conducted with a nationwide community-based older Chinese people sample, and the follow-up lasted for a relatively long period of time, which can make the estimates of transition probabilities and life expectancies in our study more accurate. However, some limitations should be noted. With respect to the methodology, the analytic strategy of this study based on the Markov process assumed that future status depends on only the present status, not on the sequence of events that preceded it, and thus, it does not account for individual heterogeneity in the disabled status history [64]. Loss to follow-up was an issue in this study, as in any other longitudinal study. However, according to Kempen and van Sonderen [45], there will be no significant problems in estimations from the CLHLS data because of the relatively low attrition from this survey [65]. In addition, new respondents conformed to the study and replenished those respondents who were lost, which can reduce this bias.

In conclusion, the results of this study reveal that rural elderly individuals live shorter lives than their urban counterparts and have a higher risk of disability. However, these individuals tend to live with better functional status than their urban counterparts, with a larger fraction of remaining life spent with less severe disability. Women have a longer life expectancy than men but spend a higher proportion of their remaining time in a disabled state. Our findings have some implications for public health officials to improve the functioning of elderly Chinese individuals. Interventions and programs should be implemented to prevent disability, slow its progression, and fill the gaps between the sexes and among regional areas. According to our study, more medical resources and health services must be allocated to rural areas to ensure that these services are accessible to the elderly in these rural areas who are more inclined to develop disabilities, especially rural women. It may be more effective to devote more attention to rural areas to help mildly disabled people who have a higher probability of recovery. At an individual level, information must be disseminated throughout the community so that rehabilitation training can be implemented as early as possible after disability occurs, which has been reported to result in better recovery and positive outcomes [66-68].

## Supplementary Materials

The Supplemenantry data can be found online at: www.aginganddisease.org/EN/10.14336/AD.2019.0121


