# Supplementary material for: Disability Transitions and Health Expectancies among Elderly People Aged 65 Years and Over in China: A Nationwide Longitudinal Study
Source: Aging Dis. 2019 Dec 1;10(6):1246–57. doi: 10.14336/AD.2019.0121 (PMC6844588; doi:10.14336/AD.2019.0121)
Supplement: Supplementary file 1 [file AD-10-6-1246-s.pdf]

SUPPLEMENTARY DATA

# Disability Transitions and Health Expectancies among Elderly People Aged 65 Years and Over in China: A Nationwide Longitudinal Study

**Chengbei Hou[1,2#], Yuan Ma[1,2#], Xinghua Yang[1,2], Lixin Tao[1,2], Deqiang Zheng[1,2], Xiangtong Liu[1,2], Xiaonan Wang[1,2], Xia Li[3], Wei Wang[4], Xianghua Fang[5*], Xiuhua Guo[1,2*]**

[1]School of Public Health, Capital Medical University, Beijing, China.
[2]Beijing Municipal Key Laboratory of Clinical Epidemiology, School of Public Health, Capital Medical University, Beijing, China
[3]Department of Mathematics and Statistics, La Trobe University, Bundoora Victoria, Australia.
[4]School of Medical Sciences and Health, Edith Cowan University, Joondalup, Perth, WA6027, Australia.
[5]Center for Evidence-Based Medicine, Xuanwu Hospital Capital Medical University, Beijing, China

© 2019. Hou C et al. Published online at http://www.aginganddisease.org/EN/10.14336/AD.2019.0121

# SUPPLEMENTARY DATA

**Supplementary Table 1.** Total life expectancy and life expectancy free from and with disability, and proportion of remaining life without or with disability at age 65-95, by gender and region, based on initial disability state.

| Age | | Initial state-Urban male | | | Initial state-Urban female | | | Initial state-Rural male | | | Initial state-Rural female | | |
|---|---|---|---|---|---|---|---|---|---|---|---|---|---|
| | | **No** | **Mild** | **Severe** | **No** | **Mild** | **Severe** | **No** | **Mild** | **Severe** | **No** | **Mild** | **Severe** |
| 65 | | | | | | | | | | | | | |
| | TLE | 15.07 (14.66,15.46) | 14.58 (14.21,14.95) | 12.63 (11.78,13.40) | 17.08 (16.72,17.51) | 16.55 (16.18,16.99) | 14.75 (13.96,15.73) | 14.72 (14.35,15.08) | 14.22 (13.87,14.57) | 12.09 (11.44,12.68) | 16.55 (16.19,16.89) | 16.02 (15.64,16.38) | 14.10 (13.42,14.75) |
| | ND | 11.14 (10.79,11.5) | 9.66 (9.16,10.15) | 6.88 (5.74,7.97) | 10.72 (10.41,11.04) | 8.95 (8.5,9.43) | 6.78 (5.66,8.21) | 10.9 (10.58,11.17) | 9.5 (9.12,9.85) | 6.66 (5.97,7.23) | 10.15 (9.87,10.44) | 8.53 (8.16,8.92) | 6.29 (5.62,7.05) |
| | PND | 73.95 (72.64,75.18) | 66.25 (63.79,68.59) | 54.5 (48.21,59.89) | 62.77 (61.47,64.01) | 54.08 (51.77,56.45) | 45.96 (39.90,52.60) | 74.06 (73.03,75.06) | 66.81 (64.85,68.44) | 55.07 (51.14,57.99) | 61.36 (60.14,62.53) | 53.21 (51.44,54.96) | 44.60 (41.31,48.43) |
| | MD | 2.50 (2.35,2.67) | 3.37 (3.1,3.65) | 2.33 (2.11,2.59) | 4.42 (4.21,4.66) | 5.48 (5.12,5.86) | 4.01 (3.71,4.38) | 2.88 (2.73,3.02) | 3.68 (3.44,3.92) | 2.83 (2.59,3.11) | 5.02 (4.8,5.24) | 6.00 (5.72,6.27) | 4.77 (4.42,5.21) |
| | PMD | 16.56 (15.63,17.50) | 23.10 (21.25,25.16) | 18.44 (17.1,20.4) | 25.89 (24.83,27.14) | 33.1 (31.00,35.24) | 27.17 (25.57,29.78) | 19.53 (18.69,20.36) | 25.88 (24.36,27.55) | 23.40 (21.7,25.32) | 30.36 (29.37,31.49) | 37.43 (35.76,39.04) | 33.85 (31.74,36.13) |
| | SD | 1.43 (1.26,1.59) | 1.55 (1.38,1.72) | 3.42 (2.84,3.98) | 1.94 (1.78,2.10) | 2.12 (1.94,2.28) | 3.96 (3.21,4.60) | 0.94 (0.85,1.04) | 1.04 (0.95,1.14) | 2.6 (2.31,2.97) | 1.37 (1.28,1.48) | 1.50 (1.38,1.63) | 3.04 (2.65,3.43) |
| | PSD | 9.48 (8.32,10.57) | 10.65 (9.48,11.80) | 27.06 (21.87,32.73) | 11.34 (10.42,12.25) | 12.82 (11.63,13.82) | 26.87 (20.66,32.23) | 6.41 (5.79,7.10) | 7.30 (6.68,8.04) | 21.53 (18.71,25.23) | 8.27 (7.73,8.99) | 9.35 (8.64,10.14) | 21.56 (18.38,24.86) |
| 75 | | | | | | | | | | | | | |
| | TLE | 9.04 (8.82,9.28) | 8.19 (7.96,8.43) | 6.47 (6.1,6.86) | 10.46 (10.23,10.74) | 9.61 (9.35,9.88) | 7.89 (7.51,8.33) | 8.70 (8.51,8.91) | 7.85 (7.64,8.08) | 6.06 (5.73,6.37) | 9.99 (9.77,10.21) | 9.19 (8.96,9.43) | 7.44 (7.07,7.76) |
| | ND | 5.72 (5.53,5.93) | 3.60 (3.35,3.85) | 1.87 (1.52,2.2) | 5.19 (5.02,5.37) | 3.03 (2.83,3.24) | 1.77 (1.43,2.09) | 5.44 (5.28,5.61) | 3.48 (3.29,3.66) | 1.82 (1.6,2.02) | 4.74 (4.59,4.89) | 2.83 (2.68,2.99) | 1.63 (1.43,1.8) |
| | PND | 63.26 (61.70,64.71) | 43.92 (41.32,46.33) | 28.82 (24.40,32.93) | 49.57 (48.08,50.88) | 31.60 (29.73,33.47) | 22.47 (18.68,25.69) | 62.58 (61.41,63.75) | 44.40 (42.42,46.22) | 30.01 (27.19,32.75) | 47.44 (46.21,48.72) | 30.84 (29.49,32.10) | 21.91 (19.75,23.77) |
| | MD | 2.15 (2.04,2.30) | 3.18 (2.99,3.37) | 1.51 (1.36,1.69) | 3.59 (3.43,3.77) | 4.58 (4.36,4.81) | 2.43 (2.21,2.7) | 2.46 (2.36,2.59) | 3.40 (3.25,3.55) | 1.94 (1.75,2.14) | 4.05 (3.90,4.20) | 4.95 (4.78,5.13) | 3.06 (2.80,3.36) |
| | PMD | 23.80 (22.65,25.16) | 38.88 (36.78,41.02) | 23.38 (21.45,25.61) | 34.28 (33.03,35.65) | 47.65 (45.72,49.59) | 30.76 (28.46,33.66) | 28.31 (27.26,29.43) | 43.36 (41.73,45.09) | 31.99 (29.61,34.50) | 40.50 (39.32,41.69) | 53.88 (52.55,55.28) | 41.10 (38.50,43.87) |
| | SD | 1.17 (1.05,1.28) | 1.41 (1.28,1.52) | 3.09 (2.84,3.36) | 1.69 (1.57,1.81) | 1.99 (1.85,2.14) | 3.69 (3.42,3.98) | 0.79 (0.73,0.86) | 0.96 (0.88,1.05) | 2.30 (2.11,2.52) | 1.20 (1.13,1.29) | 1.40 (1.30,1.51) | 2.75 (2.56,3.00) |
| | PSD | 12.94 (11.64,14.13) | 17.20 (15.64,18.69) | 47.79 (43.45,52.38) | 16.14 (15.14,17.22) | 20.76 (19.18,22.22) | 46.77 (43.1,50.45) | 9.11 (8.42,9.77) | 12.24 (11.27,13.29) | 38.00 (34.8,41.58) | 12.06 (11.38,12.85) | 15.28 (14.34,16.34) | 36.99 (33.91,40.42) |
| 85 | | | | | | | | | | | | | |
| | TLE | 4.95 (4.81,5.11) | 4.15 (3.99,4.29) | 3.16 (2.97,3.38) | 5.89 (5.72,6.06) | 5.14 (4.97,5.33) | 4.01 (3.8,4.21) | 4.66 (4.54,4.80) | 3.88 (3.76,4.00) | 2.83 (2.67,2.98) | 5.52 (5.39,5.66) | 4.84 (4.72,4.97) | 3.65 (3.48,3.79) |
| | ND | 2.60 (2.49,2.73) | 0.88 (0.8,0.96) | 0.32 (0.24,0.43) | 2.22 (2.1,2.34) | 0.69 (0.63,0.75) | 0.31 (0.23,0.36) | 2.37 (2.27,2.47) | 0.83 (0.76,0.89) | 0.30 (0.26,0.38) | 1.94 (1.84,2.05) | 0.63 (0.58,0.68) | 0.27 (0.23,0.30) |
| | | 52.56 | 21.11 | 10.22 | 37.7 | 13.48 | 7.63 | 50.93 | 21.31 | 10.74 | 35.1 | 13.06 | 7.40 |

© 2019. Hou C et al. Published online at http://www.aginganddisease.org/EN/10.14336/AD.2019.0121

| | | | | | | | | | | | | | |
|---|---|---|---|---|---|---|---|---|---|---|---|---|---|
| | PND | (50.69,54.34) | (19.44,23.02) | (7.84,13.26) | (35.87,39.34) | (12.40,14.62) | (5.86,9.04) | (49.27,52.52) | (19.79,22.9) | (9.26,13.46) | (33.44,36.79) | (12.09,14.01) | (6.29,8.29) |
| | MD | 1.54 (1.45,1.64) | 2.24 (2.12,2.36) | 0.67 (0.58,0.77) | 2.44 (2.30,2.56) | 2.97 (2.82,3.13) | 1.04 (0.92,1.17) | 1.72 (1.64,1.82) | 2.34 (2.24,2.43) | 0.90 (0.78,1.01) | 2.69 (2.59,2.80) | 3.16 (3.05,3.26) | 1.38 (1.25,1.49) |
| | PMD | 31.14 (29.25,33.1) | 54.04 (51.79,56.02) | 21.10 (18.61,23.82) | 41.38 (39.6,43.15) | 57.65 (55.92,59.35) | 25.85 (23.41,28.68) | 37.03 (35.62,38.70) | 60.26 (58.52,61.83) | 31.75 (28.08,35.40) | 48.83 (47.28,50.40) | 65.26 (63.98,66.54) | 37.93 (35.16,40.19) |
| | SD | 0.81 (0.73,0.89) | 1.03 (0.94,1.12) | 2.17 (2.02,2.33) | 1.23 (1.15,1.32) | 1.48 (1.40,1.59) | 2.67 (2.51,2.84) | 0.56 (0.52,0.60) | 0.71 (0.65,0.77) | 1.63 (1.50,1.76) | 0.89 (0.83,0.95) | 1.05 (0.98,1.11) | 2.00 (1.89,2.13) |
| | PSD | 16.30 (14.91,17.82) | 24.85 (22.99,26.79) | 68.68 (65.23,71.60) | 20.92 (19.65,22.34) | 28.86 (27.22,30.58) | 66.52 (63.84,69.45) | 12.04 (11.16,12.95) | 18.43 (17.13,19.77) | 57.51 (53.53,61.01) | 16.07 (15.12,17.05) | 21.68 (20.52,22.86) | 54.67 (52.37,57.81) |
| 95 | TLE | 2.50 (2.39,2.63) | 2.01 (1.90,2.12) | 1.53 (1.40,1.67) | 3.09 (2.96,3.23) | 2.64 (2.51,2.79) | 2.00 (1.87,2.15) | 2.31 (2.20,2.42) | 1.83 (1.73,1.91) | 1.30 (1.19,1.40) | 2.84 (2.73,2.96) | 2.43 (2.32,2.54) | 1.73 (1.62,1.84) |
| | ND | 1.10 (1.00,1.19) | 0.16 (0.14,0.18) | 0.04 (0.03,0.08) | 0.89 (0.8,0.98) | 0.12 (0.11,0.14) | 0.04 (0.03,0.06) | 0.96 (0.87,1.05) | 0.14 (0.13,0.16) | 0.04 (0.03,0.07) | 0.74 (0.65,0.83) | 0.11 (0.10,0.12) | 0.03 (0.02,0.04) |
| | PND | 43.97 (40.91,46.72) | 7.74 (6.93,8.81) | 2.70 (1.79,5.16) | 28.76 (26.05,31.39) | 4.58 (4.06,5.22) | 2.02 (1.34,2.78) | 41.65 (38.72,44.68) | 7.77 (6.86,8.80) | 2.74 (2.14,5.24) | 25.98 (23.41,28.89) | 4.42 (3.91,5.00) | 1.89 (1.46,2.35) |
| | MD | 0.94 (0.86,1.01) | 1.25 (1.16,1.34) | 0.22 (0.18,0.28) | 1.45 (1.34,1.56) | 1.62 (1.51,1.74) | 0.35 (0.29,0.40) | 1.02 (0.94,1.09) | 1.26 (1.18,1.33) | 0.31 (0.25,0.38) | 1.56 (1.47,1.65) | 1.69 (1.60,1.79) | 0.49 (0.42,0.54) |
| | PMD | 37.42 (34.77,40.24) | 62.14 (59.03,64.93) | 14.46 (11.95,17.96) | 46.92 (44.19,49.62) | 61.44 (58.79,63.92) | 17.46 (15.09,20.14) | 44.09 (41.48,47) | 69.16 (66.79,71.15) | 23.72 (19.28,28.53) | 54.86 (52.23,57.43) | 69.42 (67.49,71.49) | 28.07 (24.84,30.90) |
| | SD | 0.47 (0.41,0.53) | 0.60 (0.54,0.68) | 1.27 (1.14,1.39) | 0.75 (0.68,0.83) | 0.90 (0.82,0.99) | 1.61 (1.49,1.76) | 0.33 (0.29,0.37) | 0.42 (0.38,0.47) | 0.95 (0.85,1.05) | 0.54 (0.49,0.60) | 0.64 (0.58,0.69) | 1.21 (1.12,1.31) |
| | PSD | 18.61 (16.76,20.9) | 30.12 (27.26,33.48) | 82.83 (79.67,85.17) | 24.32 (22.06,26.80) | 33.98 (31.52,36.72) | 80.52 (77.93,82.98) | 14.26 (12.80,15.81) | 23.07 (20.81,25.37) | 73.54 (68.39,77.49) | 19.16 (17.33,20.88) | 26.16 (23.97,28.22) | 70.04 (67.18,73.30) |

Note: Data come from the Chinese Longitudinal Healthy Longevity Survey (CLHLS).
Data are n (95%CI). TLE=total life expectancy; ND=years with no disability; MD=years with mild disability; SD=years with severe disability; AD=years with any disability (MD+SD); PND=proportion of life with no disability; PMD=proportion of life with mild disability; PSD=proportion of life with severe disability; PAD=proportion of life with any disability (PMD+PSD). None=no disability; Mild=mild disability; Severe=severe disability.

© 2019. Hou C et al. Published online at http://www.aginganddisease.org/EN/10.14336/AD.2019.0121

# SUPPLEMENTARY DATA

**Supplementary Table 2.** Demographic characteristics and health status of participants and comparison between genders, urban-rural residences.

| **Variables** | **Men (N=9704)** | **Women(N=9915)** | $\chi^2$ | ***P*** | **Urban (N=7756)** | **Rural (N=11863)** | $\chi^2$ | ***P*** |
|---|---|---|---|---|---|---|---|---|
| Educational attainment, N (%) | | | 3917.82 | <0.001 | | | 761.01 | <0.001 |
| 0 year | 3401 (30.3) | 7820 (69.7) | | | 3743 (33.4) | 7478 (66.6) | | |
| 1-6 years | 4592 (73.7) | 1635 (26.3) | | | 2647 (42.5) | 3580 (57.5) | | |
| 7-9 years | 837 (79.2) | 220 (20.8) | | | 588 (55.6) | 469 (44.4) | | |
| 9 + years | 849 (81.6) | 192 (18.4) | | | 744 (71.5) | 297 (28.5) | | |
| Currently married, N (%) | 5074 (67.4) | 2450 (32.6) | 1578.55 | <0.001 | 3088 (41.0) | 4436 (59.0) | 11.56 | 0.001 |
| Currently smoking, N (%) | 3611 (82.9) | 743 (17.1) | 2508.03 | <0.001 | 1585 (36.4) | 2769 (63.6) | 22.90 | <0.001 |
| Currently drinking, N (%) | 3270 (77.5) | 952 (22.5) | 1684.49 | <0.001 | 1524 (36.1) | 2698 (63.9) | 26.55 | <0.001 |
| Enough living expenses, N (%) | 7807 (50.5) | 7649 (49.5) | 31.78 | <0.001 | 6386 (41.3) | 9070 (58.7) | 96.75 | <0.001 |
| Adequate medical service, N (%) | 8875 (50.0) | 8874 (50.0) | 21.41 | <0.001 | 7231 (40.7) | 10518 (59.3) | 113.18 | <0.001 |
| Physical exercise, N (%) | 3628 (58.1) | 2614 (41.9) | 274.59 | <0.001 | 3518 (56.4) | 2724 (43.6) | 1083.13 | <0.001 |
| Diabetes, N (%) | 228 (46.7) | 260 (53.3) | 1.50 | 0.221 | 314 (64.3) | 174 (35.7) | 128.74 | <0.001 |
| Stroke, N (%) | 603 (54.0) | 513 (46.0) | 9.90 | 0.002 | 558 (50.0) | 558 (50.0) | 54.12 | <0.001 |
| Cardiovascular disease, N (%) | 764 (44.5) | 954 (55.5) | 18.73 | <0.001 | 1016 (59.1) | 702 (40.9) | 302.49 | <0.001 |
| Disability at entry, N (%) | | | 516.21 | <0.001 | | | 152.08 | <0.001 |
| No impaired | 5314 (58.1) | 3834 (41.9) | | | 3719 (40.7) | 5429 (59.3) | | |
| Mild impaired | 3106 (42.7) | 4166 (57.3) | | | 2528 (34.8) | 4744 (65.2) | | |
| Severe impaired | 1284 (40.1) | 1915 (59.9) | | | 1509 (47.2) | 1690 (52.8) | | |

Note: Data come from the Chinese Longitudinal Healthy Longevity Survey (CLHLS).
Numbers calculated as a percentage of the nonmissing values.
The number of participants with missing values at entry was 73 for education, 2 for marital status, 14 for currently smoking, 14 for currently drinking, 4 for living expense, and 4 for medical service, 30 for physical activity, 6 for diabetes, 4 for cardiovascular disease, 5 for stroke.

© 2019. Hou C et al. Published online at http://www.aginganddisease.org/EN/10.14336/AD.2019.0121

# SUPPLEMENTARY DATA

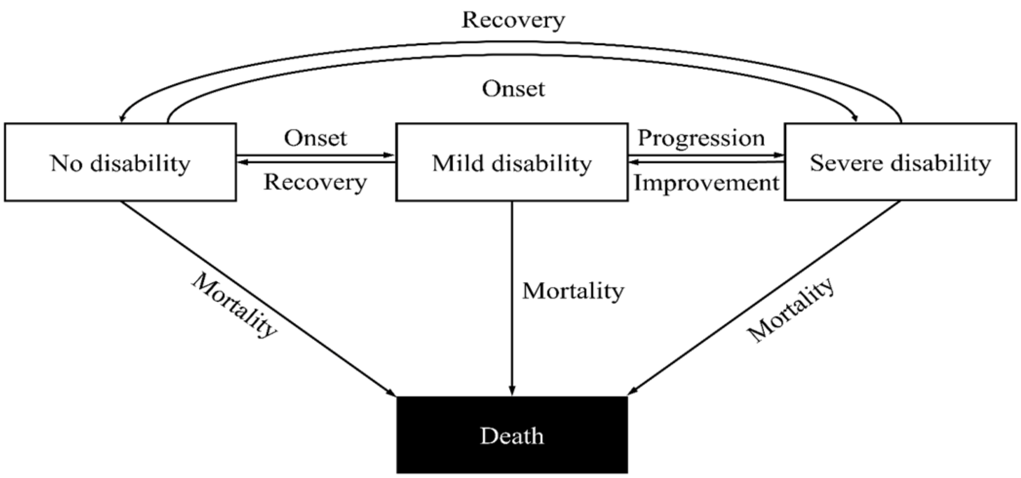


**Supplementary Figure 1. Structure of the multi-state model of disability transitions.**

© 2019. Hou C et al. Published online at http://www.aginganddisease.org/EN/10.14336/AD.2019.0121
